# Online Condition Monitoring of Bearings to Support Total Productive Maintenance in the Packaging Materials Industry

**DOI:** 10.3390/s16030316

**Published:** 2016-03-01

**Authors:** Jovan Gligorijevic, Dragoljub Gajic, Aleksandar Brkovic, Ivana Savic-Gajic, Olga Georgieva, Stefano Di Gennaro

**Affiliations:** 1 Faculty of Engineering, University of Kragujevac, Kragujevac 34000, Serbia; jovan.gligorijevic@tetrapak.com (J.G.); aleksandar.brkovic@tetrapak.com (A.B.); 2 Tetra Pak, Gornji Milanovac Packaging Materials Plant, Gornji Milanovac 32300, Serbia; 3 School of Electrical Engineering, University of Belgrade, Belgrade 11000, Serbia; 4 Faculty of Mathematics and Informatics, University of Sofia, Sofia 1000, Bulgaria; o.georgieva@fmi.uni-sofia.bg; 5 Faculty of Technology, University of Nis, Nis 18000, Serbia; ivana.savic830@gmail.com; 6 Center of Excellence DEWS, University of L’Aquila, L’Aquila 67100, Italy; stefano.digennaro@univaq.it

**Keywords:** total productive maintenance, reliability, bearings, fault diagnosis, wavelet transform, statistical pattern recognition

## Abstract

The packaging materials industry has already recognized the importance of Total Productive Maintenance as a system of proactive techniques for improving equipment reliability. Bearing faults, which often occur gradually, represent one of the foremost causes of failures in the industry. Therefore, detection of their faults in an early stage is quite important to assure reliable and efficient operation. We present a new automated technique for early fault detection and diagnosis in rolling-element bearings based on vibration signal analysis. Following the wavelet decomposition of vibration signals into a few sub-bands of interest, the standard deviation of obtained wavelet coefficients is extracted as a representative feature. Then, the feature space dimension is optimally reduced to two using scatter matrices. In the reduced two-dimensional feature space the fault detection and diagnosis is carried out by quadratic classifiers. Accuracy of the technique has been tested on four classes of the recorded vibrations signals, *i.e*., normal, with the fault of inner race, outer race, and ball operation. The overall accuracy of 98.9% has been achieved. The new technique can be used to support maintenance decision-making processes and, thus, to increase reliability and efficiency in the industry by preventing unexpected faulty operation of bearings.

## 1. Introduction

**Figure 1:**
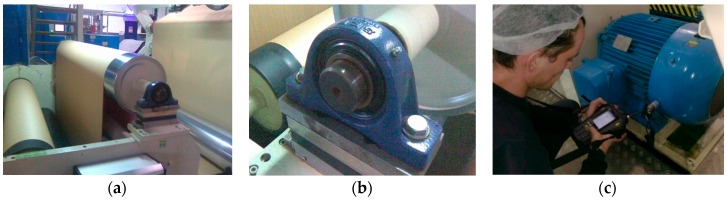
Roller (**a**); bearing (**b**); and electric motor (**c**).

**Figure 2:**
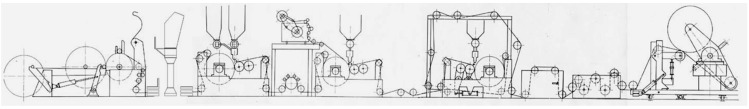
Scheme of a laminator containing around 50 rollers (courtesy of Tetra Pak).

The production processes in the packaging materials industry has to be very efficient and cost-effective. These processes usually take place under extreme conditions and high speeds that require a high level of both reliability and safety. In order to further increase its competitiveness the packaging materials industry needs to deploy advanced maintenance strategies and solutions for improved reliability of its production equipment, as well as safety of its production processes. Total Productive Maintenance (TPM) is one such strategy that is usually performed at the plant level. As such, it plays an increasingly important role in order to achieve a reliable, safe, and efficient operation of production machines. TPM aims to optimize the entire maintenance program and define optimal measures to be implemented on each of the machines. Manufacturing of paper-based packaging materials usually includes three key production processes which are printing, laminating, and slitting, in that order. All of these processes take place under extreme conditions and high speeds of up to 1000 m/min that requires a high level of reliability and safety. Rollers, together with their supporting bearings and electric motors, as shown in Figure 1 and Figure 2, are the most common components of these production machines. Bearings operate under high loading and severe conditions and their faults represent one of the foremost causes of failures.

**Figure 3:**
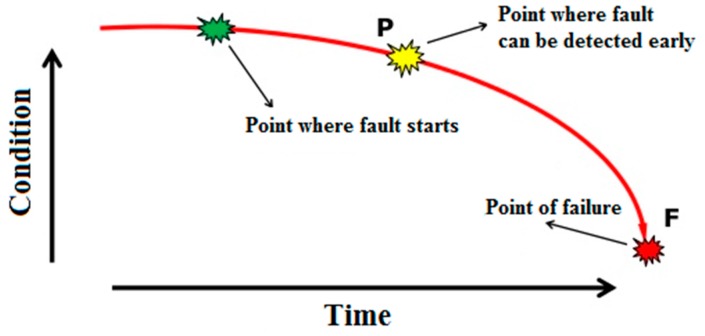
Predictive maintenance Potential to Functional Failure (P-F) curve.

**Figure 4:**
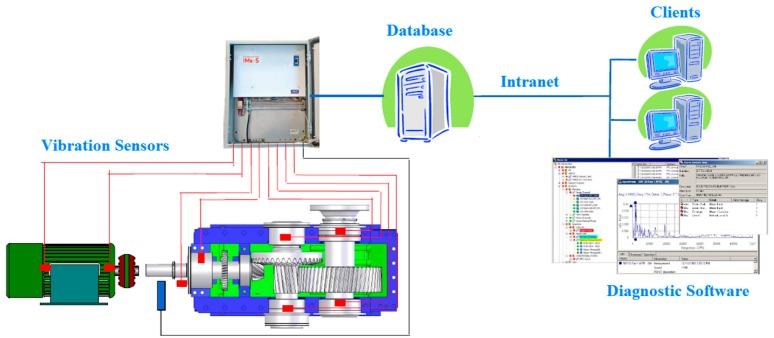
Online vibration monitoring system (courtesy of SKF).

As shown in Figure 3, bearing faults often occur gradually. Defective bearings generate various forces causing high amplitude vibrations. Therefore, it is very important to avoid deteriorating conditions, degraded reliability, and unexpected failures of bearings. Based on TPM analysis performed on the laminator at the Tetra Pak packaging materials plant in Gornji Milanovac, Serbia it has been concluded that, for most of bearings, a regular check of vibration levels every two weeks by a portable, handheld data collector and analyzer can ensure their reliable operation and early detection of bearing faults [1]. However at a few critical points it has been proposed to do more frequent checks that becomes resource-demanding and not so optimal anymore. Therefore, installation of an online vibration monitoring system including deployment of an automated technique for early fault detection and diagnosis (FDD) in bearings is needed. Such a system saves the time of maintenance technicians and enables an objective, reliable, and faster detection and diagnosis of bearing faults. Since vibration sensors and data acquisition systems are already available in the market, the Tetra Pak plant has designed an online vibration monitoring system as presented in Figure 4. The online vibration sensors will continually monitor the condition of a few critical bearings whose failure in the past caused stoppages of the entire production for a couple of hours, resulting in a significant loss. At the same time, the plant also initiated development of a new automated technique for early FDD in bearings to be tested and potentially deployed in this plant once all the equipment is installed. This paper focuses on development of such a technique.

Even though there are some techniques for FDD in bearings based on force measurements [2] most of them that have emerged in recent years are based on vibration signal analysis. Generally, an FDD can be decomposed into three steps: data acquisition, feature extraction, and classification. An effective feature extraction as the key step represents a mapping of vibration signals from their original measured space to the feature space which contains more valuable information for FDD. Even though time-domain features, e.g., peak, mean, root mean square, and variance, have also been employed as input features to train a bearing FDD classifier, the fast Fourier transform (FFT) is the most widely applied and established feature extraction methods [3]. However, the techniques based on FFT are not suitable for analysis of non-stationary signals. Since vibration signals often contain non-stationary components, for a successful FDD it is very important to reveal such information as well. Thus, a supplementary technique for non-stationary signal analysis is necessary. Time-frequency techniques, e.g., the Wigner-Ville distribution (WVD) [4] and the short-time Fourier transform (STFT) [5] also have their own disadvantages. The WVD bilinear characteristic causes interference terms in the time-frequency domain while the STFT results in a constant resolution for all frequencies having in mind that it uses the same window size for the analysis. The wavelet transform resolves all these deficiencies. It ensures a good frequency resolution and low time resolution for low-frequency components while, for high-frequency components, it provides low-frequency resolution and good time resolution. Therefore, the wavelet transform is widely applied in the vibration signal analysis and feature extraction for bearing FDD [6,7]. Classification, as the next step, directly depends on previously extracted features, *i.e.*, there is no classifier which can make up for the information lost during the feature extraction. As in the case of the feature extraction, we can come across a wide range of classifiers used for FDD in bearings. The classifiers based on artificial neural networks [8,9,10] and fuzzy logic [11,12] demonstrated a very reliable classification. However, the disadvantage of the mentioned classification techniques is that they require the availability of a very large training set. They also have a large number of parameters to be selected and adjusted in order to obtain acceptable results [13]. Therefore, there is a strong need to make the classification process simpler, faster, and accurate using as few features and parameters as possible.

**Figure 5:**
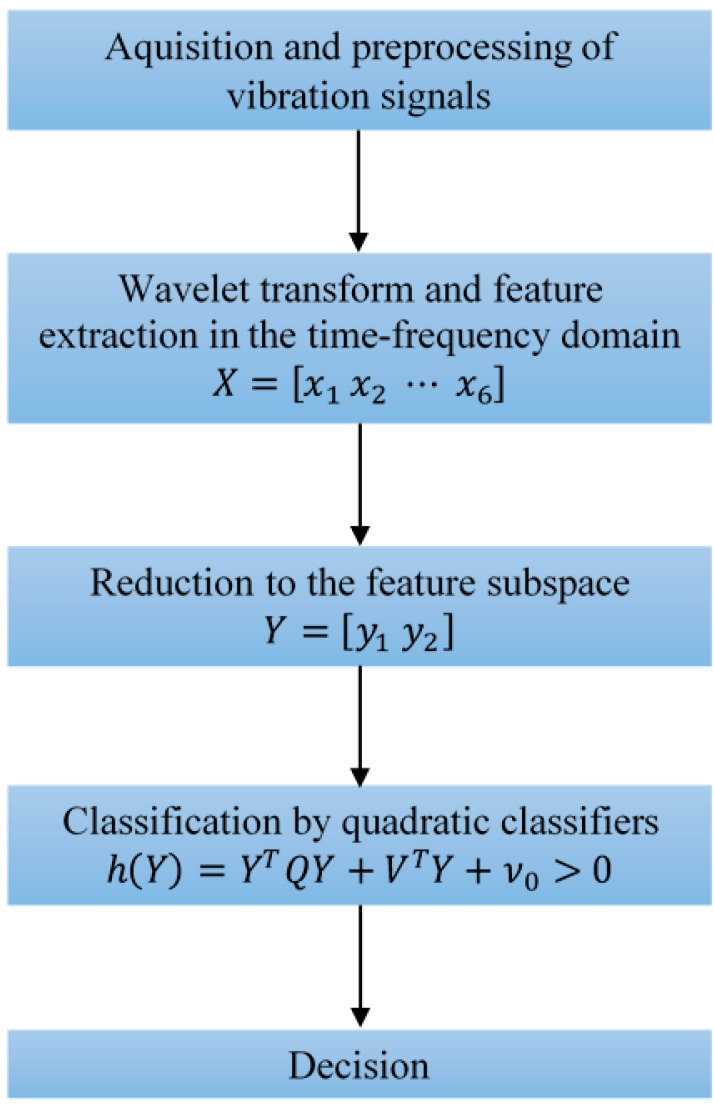
Flowchart of the new technique for early FDD.

In this paper a new technique for early FDD is proposed. As shown in Figure 5 it has a few steps. The first one is acquisition of signals, as well as their preprocessing, which includes normalization and segmentation. The vibration signals are normalized to zero mean and unit variance in order to minimize impact of their magnitude on accuracy. Even though the signals are recorded in real-time at the sampling frequency of 12,000 Hz they are further processed in subsequent segments of 1024 samples each, which enables faster execution of the entire technique and also, at the same time, captures all the fault frequencies of interest. In the next step, and after the wavelet transform of vibration signals, six representative features, *i.e.*, the standard deviation of the wavelet coefficients in six sub-bands, are extracted in the time-frequency domain. The standard deviation is used as a measure of the average and relative energy in each sub-band since the vibration signals were previously normalized to zero mean and unit variance. In the third step the feature space dimension is optimally reduced to two using scatter matrices and keeping in mind their rank. The dimension reduction to two also enables much better visualization of classification results. In the fourth step, a total of three quadratic classifiers are designed [14], one for detection and the other two for diagnosis of bearing faults. Based on the classification results in the final step a decision about bearing state is made. Using this new approach, the overall complexity of FDD is decreased and, at the same time, a very high accuracy maintained compared with already available techniques which deploy more complex training algorithms.

## 2. Materials and Methods

### 2.1. Acquisition and Preprocessing of the Vibration Signals

**Figure 6:**
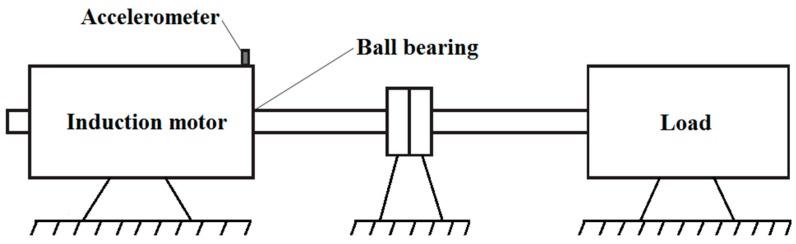
Experimental system.

Before deployment of the new technique into a real production environment, its capability was tested using the vibration data obtained from the CWRU Bearing Data Center [11]. This vibration data has become a standard reference in the field of FDD in bearings and enables an objective comparison of different techniques developed so far. A ball bearing SKF 6205-2RS JEM (SKF, Gothenburg, Sweden) was installed in a motor-driven system as presented in Figure 6. The bearing fault frequencies as a multiple of shaft speed are as follows [15]:

Ball pass frequency, outer race: 5.415;
Ball pass frequency, inner race: 3.585;
Fundamental train frequency (cage speed): 0.3983; and
Ball (roller) spin frequency: 2.357.

An accelerometer with a bandwidth up to 5000 Hz and a 1 V/g output was used to acquire the vibration signals from the bearing. Acceleration was measured in the vertical direction on the housing of the drive-end bearing. The sampling rate of 12,000 Hz is sufficient, keeping in mind that the frequency content of interest does not exceed 5000 Hz. In total, four sets of data were obtained and used:

1. Under normal conditions;
2. With inner race faults;
3. With ball faults; and
4. With outer race faults.

**Figure 7:**
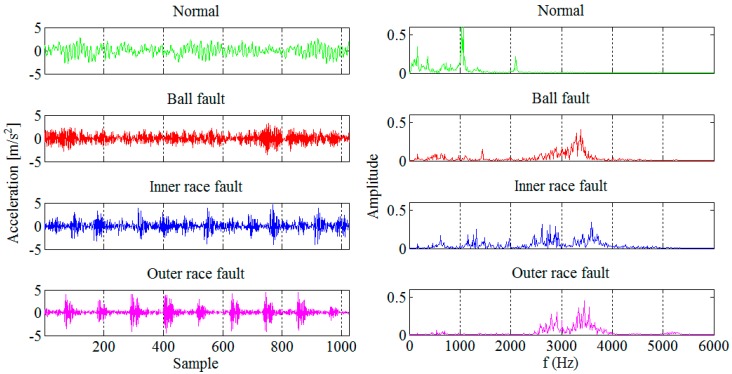
Segments of the vibration signals collected from four different conditions of the ball bearing (**left**) and their frequency spectra (**right**).

The faults with diameter from 0.007 to 0.40 inches and depth of 0.011 inches were introduced separately at the bearing elements. The bearing was tested under different loads, *i.e.*, 0, 1, 2, and 3 hp, while the shaft rotating speeds were 1730, 1750, 1772, and 1797 rpm. Only the smallest fault diameter was selected for this study since we were interested in an early FDD. In order to make the entire technique more robust and less dependent on magnitude, the recorded vibration signals were normalized to zero mean and unit variance. The vibration signals collected from each of the four different conditions are divided into 256 segments of 1024 sample each, as shown in Figure 7. The segment length was chosen such that it enables real-time implementation of the technique in an industrial environment and also capture the bearing fault frequencies of interest. In total 1024 segments were used, 512 for design and 512 for testing of the new technique for early FDD in bearings. Based on the frequency spectra given in Figure 7 we can notice that it is easy to detect a bearing fault while its diagnosis is not so straightforward.

### 2.2. Wavelet Transform

As we already know, a signal can be represented as linear combination of basic functions. A unit impulse function with limited power is limited and non-zero mean is the basic function in the time domain. In the frequency domain, the role of a basic function is assigned to the sinusoidal function with infinite power and zero mean. When using the wavelet transform to transform the signal from the time domain to the time-frequency domain, the basic function is the wavelet. The wavelet is a function of limited power and zero mean [16], and for which the following is valid:

(1) $$\overset{\infty }{\underset{n=-\infty }{\sum }}{\left|\psi \left[n\right]\right|}^{2}<\infty ,\overset{\infty }{\underset{n=-\infty }{\sum }}\psi \left[n\right]=0$$

The wavelet can be moved in time for b samples and scaled by the so-called dilation parameter a. In such a case it is given by:

(2) $$\psi_{ab}\left[n\right]=\frac{1}{\sqrt{a}}\psi \left[\frac{n-b}{a}\right]$$

**Figure 8:**
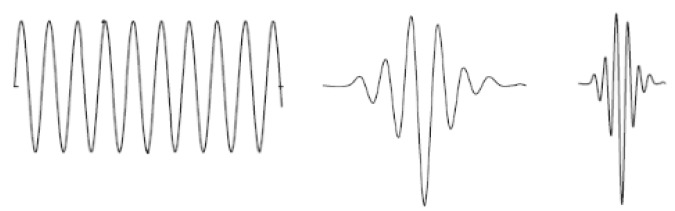
Sinusoid and two wavelets with different width.

If the dilation parameter changes, the basic wavelet (a=1) changes its width and, thus, spreads (a>1) or contracts (0≤a<1) in the time domain as shown in Figure 8. In the analysis of non-stationary signals, such a possibility represents a significant advantage, considering the fact that wider wavelets are useful for extraction of slower changes, *i.e.*, low-frequency components, while narrower wavelets are useful for extraction of faster changes, *i.e.*, high-frequency components. Following the selection of a and b it is possible to transform segments of the signal x[k] of N samples, and calculate the wavelet transform coefficients in the following way:

(3) $$w_{ab}\left[n\right]=\overset{N}{\underset{\tau =1}{\sum }}x\left[\tau \right]\psi_{ab}\left[n-\tau \right],1\leq n\leq N$$

Only those frequencies which are within the wavelet frequency band $\psi_{ab}\left[n\right]$ are extracted, *i.e.*, the signal is filtered by the wavelet $\psi_{ab}\left[n\right].$Using the wavelet coefficients, the original signal can be reconstructed that is inverse wavelet transform. It is also possible to independently reconstruct both filtered and rejected parts of the signal by the wavelet $\psi_{ab}\left[n\right]$ using the so-called detail and approximation coefficients, respectively, which are, of course, a function of the transformation coefficients $\psi_{ab}\left[n\right].$

Parameters a and b can be continuous. However this is not so practical since the signal can be transformed and reconstructed by using smaller number of wavelets, *i.e.*, a limited number of discrete values of a and b. It is known as the discrete wavelet transform (DWT) where parameters a and b are the powers of 2 and, in that case, frequency bands do not overlap each other. The dilation parameter *a*, as the power of 2, at each subsequent higher level of transformation, doubles in value in comparison to the value from the previous level. Thus, the signal frequency band from the previous level is split into two halves at every next level, into a higher band which contains finer changes, or details, and a lower band, which is an approximation of the signal from the previous level. This technique is known as the wavelet packet decomposition. We apply the five-level wavelet decomposition of the vibration signals recorded with a sampling frequency of 12,000 Hz that results into the following six frequency sub-bands $A_{5}$ 0–187.5 Hz, $D_{5}$ 187.5–375 Hz, $D_{4}$ 375–750 Hz, $D_{3}$ 750–1500 Hz, $D_{2}$ 1500–3000 Hz, and $D_{1}$ 3000–6000 Hz. After the wavelet decomposition, the standard deviation of the obtained wavelet coefficients in each sub-band is extracted as representative feature that results into a six-dimensional feature vector $X={\left[x_{1}\;x_{2}\;\cdots \;x_{6}\right]}^{T}$ for each of the analyzed segments.

### 2.3. Dimension Reduction in the Feature Space

**Figure 9:**
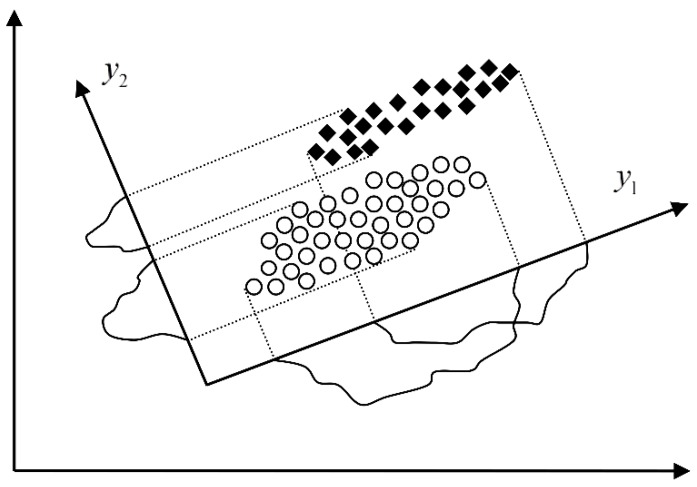
Dimension reduction in the feature space.

Let a feature vector $X={\left[x_{1}\;x_{2}\;\cdots \;x_{n}\right]}^{T}$ be transformed into $Y={\left[y_{1}\;y_{2}\;\cdots \;y_{n}\right]}^{T}=A^{T}X$ where A is an *n*-dimensional transformation matrix. Mapping of X into the space which is made up by the eigenvectors $\Phi_{1},\;\Phi_{2},\ldots ,\Phi_{n}$ of its covariance matrix $\Sigma_{X}$ is known as the principal component analysis (PCA) [17]. When reducing the feature space dimension using the PCA the performance of each feature $x_{1},\;x_{2},\ldots ,\;x_{n}$ is characterized by its eigenvalue $\lambda_{1},\;\lambda_{2},\;\ldots ,\lambda_{n}$, respectively. Thus, by rejecting features, we should first reject those with the smallest eigenvalue. In other words, the rejected features have the smallest variance in the new feature space. In the case shown in Figure 9 where the dimension is reduced from two to one, the mapped feature $y_{2}$ would be rejected based on the PCA as less informative even though it has better discriminatory potential than $y_{1}$.

Unlike the PCA, the dimension reduction based on scatter matrices [14] is more interesting in this work since it also takes into consideration classification as one of the purposes of the dimension reduction. Let L be the number of classes to be classified and $M_{i}$ and $\Sigma_{i},\;i=1\cdots L$ their mean vectors and covariance matrices. Then the within-class scatter matrix is given as:

(4) $$S_{W}=\overset{L}{\underset{i=1}{\sum }}P_{i}E\left\{\left(X-M_{i}\right){\left(X-M_{i}\right)}^{T}/\omega_{i}\right\}=\overset{L}{\underset{i=1}{\sum }}P_{i}\Sigma_{i}$$

while the between-class scatter matrix is defined as:

(5) $$S_{B}=\overset{L}{\underset{i=1}{\sum }}P_{i}\left(M_{i}-M_{0}\right){\left(M_{i}-M_{0}\right)}^{T}$$

$M_{0}$ is the joint vector of mathematical expectation for all the classes together, *i.e.*:

(6) $$M_{0}=E\left\{X\right\}=\overset{L}{\underset{i=1}{\sum }}P_{i}M_{i}$$

In addition the mixed scatter matrix can be given as:

(7) $$S_{M}=E\left\{\left(X-M_{0}\right){\left(X-M_{0}\right)}^{T}\right\}=S_{W}+S_{B}$$

Then the problem of dimension reduction is reduced to the identification of the n×m transformation matrix A which maps the n-dimensional vector X into the m-dimesnional random vector $Y=A^{T}X$ and also maximizes the criteria $J=tr\left(S_{W}^{-1}S_{B}\right)$. This criteria is invariant to non-singular linear transformations and results into transformation matrix that takes the following form:

(8) $$A=\left[\Psi_{1}\;\Psi_{2}\;\cdots \;\Psi_{m}\right]$$

where $\Psi_{i}$, i=1,…,m are the eigenvectors of $S_{W}^{-1}S_{B}$ which correspond to its m greatest eigenvalues. The dimension reduction based on scatter matrices applied to the case shown in Figure 9 would result into selection of the mapped feature $y_{2}$. Obviously it is a much better choice than $y_{1}$ selected by the PCA in terms of more accurate classification as the main goal of the dimension reduction. Since the rank of $S_{W}^{-1}S_{B}$ is at most L−1 (L is the number of classes) we can reduce the dimensionality to, at most, L−1. Having in mind that in our application we are going to deal with a maximum of three classes the dimension will be reduced to two.

### 2.4. Design of Quadratic Classifiers

Following the dimension reduction to two, as a next step we design quadratic classifiers in order to detect and diagnose different bearing faults. Quadratic classifiers are known as very robust solutions to the classification problems whose statistical features change over time [14]. In addition, they also provide visual insight into the classification problem. Quadratic classifiers to be designed in the two-dimensional feature subspace $Y={\left[y_{1}\;y_{2}\right]}^{T}$ can be defined by the following equation:

(9) $$h\left(Y\right)=Y^{T}QY+V^{T}Y+\nu_{0}=\left[y_{1}\;y_{2}\right]\left[\begin{array}{cc} q_{11} & q_{12}\\ q_{21} & q_{22} \end{array}\right]\left[\begin{array}{c} y_{1}\\ y_{2} \end{array}\right]+\left[\nu_{1}\;\nu_{2}\right]\left[\begin{array}{c} y_{1}\\ y_{2} \end{array}\right]+\nu_{0}$$

The matrix Q, vector V and scalar $\nu_{0}$ are the unknowns to be optimally determined. Equation (9) can be represented in a linear form as:

(10) $$h\left(Y\right)=\left[q_{11}\;q_{12}\;q_{22}\;\nu_{1}\;\nu_{2}\right]\left[\;\begin{array}{c} y_{1}^{2}\\ 2y_{1}y_{2}\\ \begin{array}{c} y_{2}^{2}\\ y_{1}\\ y_{2} \end{array} \end{array}\right]+\nu_{0}=V_{z}^{T}Z+\nu_{0}$$

In order to achieve as large as possible between-class and as short as possible within-class scattering we have selected the following function as the optimization criterion [14]:

(11) $$f=\frac{P_{1}{\eta_{1}}^{2}+P_{2}{\eta_{2}}^{2}}{P_{1}{\sigma_{1}}^{2}+P_{2}{\sigma_{2}}^{2}}$$

where $P_{1}$ and $P_{2}$ are probabilities and:

(12) $$\eta_{l}=E\left\{h\left(Z\right)/\omega_{l}\right\}=E\left\{V_{z}^{T}Z+\nu_{0}/\omega_{l}\right\}=V_{z}^{T}M_{l}+\nu_{0}$$

(13) $${\sigma_{l}}^{2}=var\left\{h\left(Z\right)/\omega_{l}\right\}=var\left\{V_{z}^{T}Z+\nu_{0}/\omega_{l}\right\}=V_{z}^{T}\Sigma_{l}V_{z}$$

$M_{l}$ and $\Sigma_{l\;}$ are the mean vectors and covariance matrices, respectively, of the vector Z for each of classes which should be classified. After optimization of the function f, the optimal vector $V_{z}$ and, thus, the optimal matrix Q and vector V, gets the following form:

(14) $$V_{z}=\left[\;\begin{array}{c} q_{11}\\ q_{12}\\ \begin{array}{c} q_{22}\\ \nu_{1}\\ \nu_{2} \end{array} \end{array}\right]={\left[P_{1}\Sigma_{1}+P_{2}\Sigma_{2}\right]}^{-1}\left(M_{2}-M_{1}\right)$$

while the optimal scalar is:

(15) $$\nu_{0}=-V_{z}^{T}\left(P_{1}M_{1}+P_{2}M_{2}\right)$$

which finishes the design procedure of quadratic classifiers. Based on the designed classifiers a decision about bearing state can be made as it will be demonstrated in the next section.

## 3. Results and Discussion

In order to extract representative features, and before we apply the wavelet transform, it is necessary to choose both the basic wavelet type and the number of resolution levels into which the vibration signal segments will be decomposed. After analysis of a few types of the basic wavelets, the fourth-order Daubechies wavelet was selected. It demonstrated the best discriminatory potential and resulted into the fault detection accuracy that was higher by 2%–5% compared with other basic wavelets. This type of wavelet has very good localizing properties in the time-frequency domain due to its shape and frequency characteristics [18] and, thus, is very suitable for this particular application. Following the five-level wavelet decomposition, the standard deviation of the obtained wavelet coefficients in each sub-band is extracted as representative feature in the time-frequency domain. Note that the standard deviation is used here to quantify the average and relative energy in each sub-band because the vibration signals were previously normalized to zero mean and unit variance. In total, six features were extracted for each of 1024 analyzed segments. So now each original segment recorded in the time domain is represented by its feature vector $X={\left[x_{1}x_{2}\cdots x_{6}\right]}^{T}$ that consists of standard deviations of the wavelet coefficients in six sub-bands. The extracted features for all four classes together with their statistics are given in Table 1.

**Figure 10:**
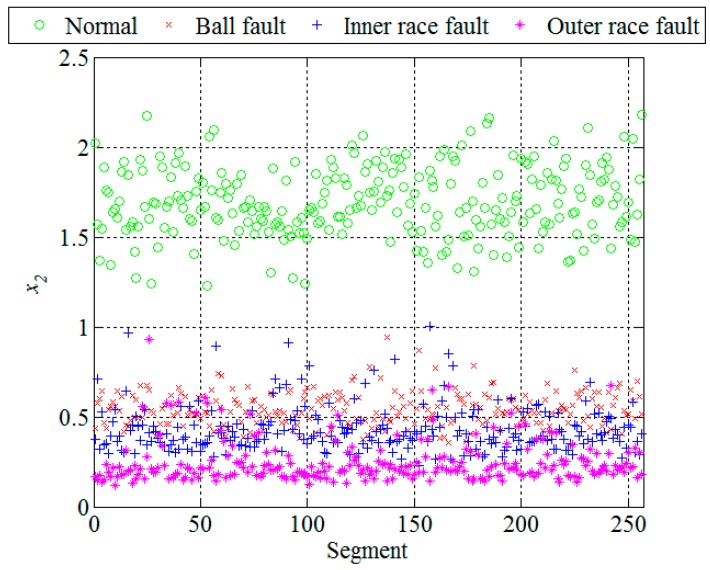
Standard deviation of the wavelet coefficients in the sub-band $D_{5}$.

**Figure 11:**
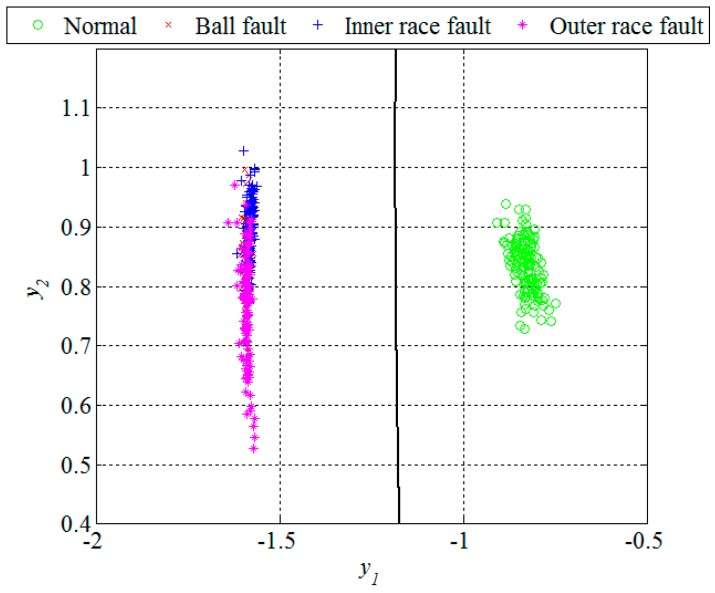
Dimension reduction and classification for the fault detection, segments of the design set.

**Figure 12:**
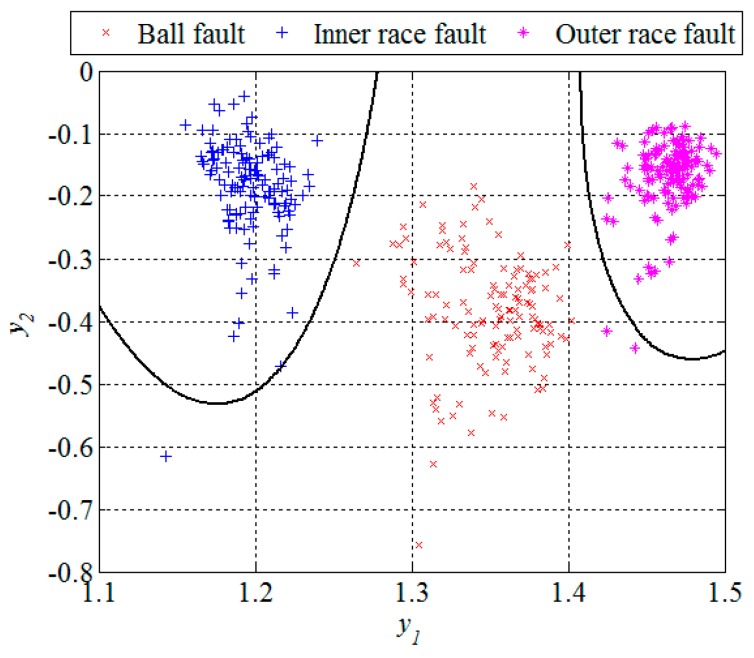
Dimension reduction and classification for the fault diagnosis, segments of the design set.

Obviously, in the presence of a bearing fault there is a shift in energy in the vibration signals from lower to higher sub-bands. In Figure 7 and Figure 10, and Table 1, it can be noticed that most of the extracted features have a certain potential for the fault detection but not for the fault diagnosis. Therefore it is necessary to find their optimal combination in order to achieve a better separability between different classes of bearing condition. That is usually done by a mapping of the existing feature space into a new one whose dimension can be reduced without any significant loss of information, which makes the classification process much simpler. Although the PCA is one of the most widely used techniques for reduction of the feature space dimension in this paper we apply the technique based on scatter matrices [14] since it is more suitable for classification problems, as described in Section 2.3. At first, we reduce the feature space dimension to enable the fault detection and then repeat the same procedure in order to diagnose the detected fault as shown in Figure 11 and Figure 12. In this way separabilty between different classes is increased compared with Figure 10. After the dimension reduction, we designed suitable quadratic classifiers following the procedure described in Section 2.4, which is also the last step in design of the new technique for early FDD. The first quadratic classifiers shown in Figure 11 separates normal from faulty condition, i.e., performs the fault detection, while the other two quadratic classifiers shown in Figure 12 are able to separate all three different bearing faults from each other and, thus, perform the fault diagnosis.

The classification results can be given by a confusion matrix which compares the classification results with ground truth information, as is shown in Table 2. Based on Figure 12 and the confusion matrix, we can conclude that all the segments from the ball fault class were correctly classified. However, the remaining two classes contained in total three segments which were incorrectly classified, *i.e.*, classified as they belong to the ball fault class. Statistical performances, such as sensitivity, specificity, and accuracy of the new technique are estimated based on these classification results. The sensitivity is defined as a ratio between the number of correctly classified segments and the total number of the segments for each of the classes separately. The specificity is also calculated for each of these three classes separately and represents the ratio between the number of correctly classified features of the other two classes and the total number of the segments of these two classes. The accuracy is calculated as the ratio between the total number of correctly classified segments and the total number of the segments in all three classes together. For segments of the design set, the accuracy of 99.2 was achieved, while other statistical performances are shown in Table 3.

**Figure 13:**
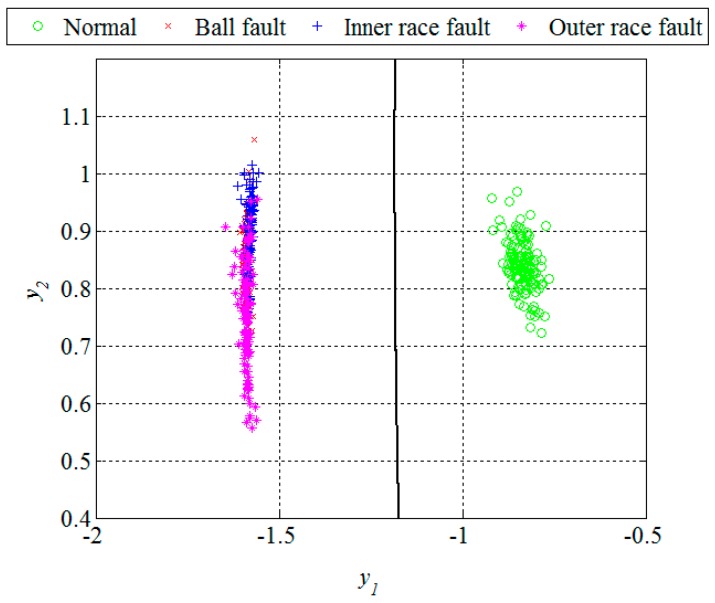
Dimension reduction and classification for the fault detection, segments of the testing set.

**Figure 14:**
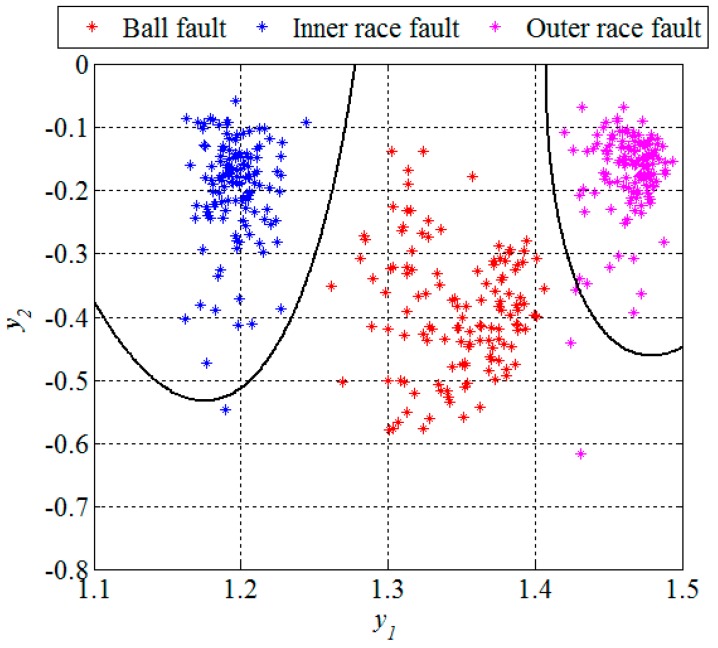
Dimension reduction and classification for the fault diagnosis, segments of the testing set.

Unlike in the previous two figures, where the design set of 512 segments is shown, Figure 13 and Figure 14 show the remaining set of 512 segments used to test the performance of the new technique for early FDD as well. The confusion matrix and statistical performances for the testing set are given in Table 4 and Table 5, respectively. The total accuracy of the new technique for early FDD in bearings is 98.9%. Usually, quadratic classifiers are robust and do not result in overtraining when the number of estimated parameters is much less than the number of analyzed samples, as in this case. However, it is a good practice to cross-validate these quadratic classifier in order to ensure its stability. A five-fold cross-validation was performed and it resulted in cross-validation loss, *i.e.*, the error of the out-of-fold samples, of 1.4%. Even though it is slightly higher than the classification error of 1.1% it gives a confidence that the classifier is reasonably stable.

Taking into account the results of other techniques tested on the same vibrations signals [18], the new technique demonstrated very good performance, which is either better than, or comparable to, other available techniques which usually deploy much more complex algorithms, as can be seen from Table 6. In addition, in this work only the segments with the smallest fault diameter were used because we were interested in incipient FDD. It should also be emphasized that the vibration signals were normalized before further processing. In that way we managed to overcome one of the main disadvantages of other techniques in terms of application in a real production environment since most of them also depend on the amplitude of the vibration signals. However, the amplitude has been found as unreliable in real applications since it varies even with healthy bearings, e.g., depending on their load. The vibration data obtained from the CWRU Bearing Data Center have quite a high signal to noise ratio, as can be noticed in Figure 7. In order to successfully deal with much heavier noise, deployment of the technique in a real production environment at Tetra Pak will most probably require extraction of some additional features, such as those obtained based on the kurtogram [19] and the sparsogram [20] of the vibration signals. The kurtogram, based on the short time Fourier transform or finite impulse response filters, limits its accuracy in extracting transient characteristics from a noisy signal. However, the improved kurtogram, which incorporates more precise filters, such as those based on the wavelet packets [21], can filter out noise much better and precisely match the fault characteristics.

## 4. Conclusions

In order to further increase reliability and safety of production machines in the packaging materials industry it is necessary to deploy an advanced techniques for automated early fault detection and diagnosis. In this paper we described such a technique to be used in rotating-element bearings as the most common components of production machines in the industry. The new technique consists of several steps. At first, the recorded vibration signals are normalized and segmented. Then, following the five-level wavelet decomposition of the vibration signals, the standard deviations of the wavelet coefficients from six sub-bands are extracted as representative features. In order to simplify classification, the dimension of the original six-dimensional feature space is reduced to two. The new technique demonstrated a very high accuracy of 98.9% that is either better than, or comparable to, other available techniques presented in Table 6 which, in most cases, require a large training set and have a large number of parameters to be selected and adjusted in order to obtain acceptable results. Special attention has been paid to robustness of the new technique, not only during the feature extraction and the dimension reduction, but also during the classification process that resulted in the choice of quadratic classifiers known for both their simplicity and a high level of robustness in the applications of this type. Quadratic classifiers have also possibility to visualize the classification results in two-dimensional space. As future work, we plan to test the new technique in a real production environment at Tetra Pak.

## Figures and Tables

**Table 1:** Standard deviations of the wavelet coefficients in six sub-bands of interest.

Feature	Sub-Band	Normal		Ball Fault		Inner Race Fault		Outer Race Fault	
		μ	σ	μ	σ	μ	σ	μ	σ
$x_{1}$	$A_{5}$ 0–187.5 Hz	2.705	0.585	0.795	0.383	0.728	0.467	0.473	0.484
$x_{2}$	$D_{5}$ 187.5–375 Hz	1.694	0.193	0.545	0.093	0.431	0.126	0.247	0.116
$x_{3}$	$D_{4}$ 375–750 Hz	1.288	0.106	0.636	0.092	0.750	0.058	0.302	0.063
$x_{4}$	$D_{3}$ 750–1500 Hz	1.847	0.199	0.525	0.087	0.919	0.044	0.243	0.056
$x_{5}$	$D_{2}$ 1500–3000 Hz	0.800	0.033	1.239	0.052	1.267	0.057	1.255	0.097
$x_{6}$	$D_{1}$ 3000–6000 Hz	0.220	0.025	1.041	0.035	0.951	0.037	1.083	0.055

**Table 2:** Confusion matrix, segments of the design set.

Fault Type (Input/Output)	Ball Fault	Inner Race Fault	Outer Race Fault
Ball fault	128	1	2
Inner race fault	0	127	0
Outer race fault	0	0	126

**Table 3:** Statistical performances, segments of the design set.

Fault Type	Statistical Performances (%)		
	Sensitivity	Specificity	Accuracy
Ball fault	100.0	98.4	99.2
Inner race fault	99.2	99.2	
Outer race fault	98.4	99.6	

**Table 4:** Confusion matrix, segments of the testing set.

Fault Type (Input/Output)	Ball Fault	Inner Race Fault	Outer Race Fault
Ball fault	128	1	3
Inner race fault	0	127	0
Outer race fault	0	0	125

**Table 5:** Statistical performances, segments of the testing set.

Fault Type	Statistical Performances (%)		
	Sensitivity	Specificity	Accuracy
Ball fault	100.0	98.4	98.9
Inner race fault	99.2	98.8	
Outer race fault	96.6	99.6	

**Table 6:** Comparison between a number of other techniques. Support vector regressive (SVR), local characteristic-scale decomposition (LCD), support vector machine (SVM), adaptive neuro-fuzzy inference systems (ANFIS), higher order statistics analysis (HOSA), principal components analysis (PCA), artificial neural networks (ANN), improved ant colony optimization (IACO), radial basis function (RBF), intrinsic mode functions (IMFs), ensemble empirical mode decomposition (EEMD), inter-cluster distance (ICD), Hilbert-Huang transform (HHT), window marginal spectrum clustering (WMSC), improved particles warm optimization (IPSO), least squares support vector machine (LSSVM), symbolic aggregate approximation (SAX), discrete wavelet transform (DWT), hierarchical transition matrix model (HTMM).

Reference	Feature Extraction	Classification	Accuracy (%)
[22]	Wavelet packets	C1: SVR C2: “one-against-one” SVM	C1: 100.00 C2: 97.90
[23]	LCD + fuzzy entropy	ANFIS	100.00
[24]	HOSA + PCA	“one-against all” SVM	96.98
[25]	Time–frequency domain	ANN	93.00
[26]	Time domain	IACO-SVM	97.50
[27]	Time- and frequency-domains	SVM	98.70
[28]	Time- and frequency-domains	C1: SVM with RBF kernel C2: Wavelet-SVM	C1: 91.25 C2: 97.50
[29]	IMFs decomposed by EEMD	SVM with parameter optimized by ICD	97.91
[30]	HHT and WMSC	SVM	100.00
[31]	IMFs decomposed by EMD	IPSO-LSSVM	97.50
[32]	SAX and DWT	HTMM	99.90
